# Incidence and Risk Factors of Dysphagia After Cardiac Surgery: A Scoping Review

**DOI:** 10.3390/jcm14124279

**Published:** 2025-06-16

**Authors:** Christos Kourek, Vania Labropoulou, Emilia Michou, Stavros Dimopoulos

**Affiliations:** 1 Clinical Ergospirometry, Exercise & Rehabilitation Laboratory, 1st Critical Care Medicine Department, Evangelismos Hospital, National and Kapodistrian University of Athens, 10676 Athens, Greece; chris.kourek.92@gmail.com (C.K.); vanialabropoulou@hotmail.com (V.L.); 2 Department of Speech Language Therapy, School of Health Rehabilitation Sciences, University of Patras, 26504 Patras, Greece; emiliamichou@upatras.gr; 3 Centre for Gastrointestinal Sciences, Faculty of Biology, Medicine and Health, University of Manchester and the Manchester Academic Health Sciences Centre, Manchester M1 39PL, UK; 4 Cardiac Surgery ICU, Onassis Cardiac Surgery Center, 17674 Kallithea, Greece

**Keywords:** dysphagia, cardiac surgery, risk factors, post-operative complications, swallowing disorders

## Abstract

Dysphagia is a serious complication following cardiac surgery, associated with increased morbidity, prolonged hospitalization, and higher healthcare costs. Variability in the incidence and risk factors highlights the need for consolidated evidence. This scoping review aimed to analyze the incidence of dysphagia after cardiac surgery and identify the associated risk factors. A search was conducted in the PubMed, Embase, Web of Sciences, and PEDro databases for observational studies reporting dysphagia incidence and risk factors in adult cardiac surgery patients. The Newcastle–Ottawa Scale was used to assess the studies’ quality and out of 2920 studies identified, 15 met the inclusion criteria for inclusion in this review. Dysphagia incidence ranged from 2.7% to 60%, with higher rates observed when objective assessments such as FEES or VFSS were employed. Key risk factors included advanced age, prolonged intubation, cerebrovascular events, and complex operative procedures. Post-operative dysphagia was linked to complications like aspiration pneumonia, prolonged ICU/hospital stays, and increased healthcare costs. In conclusion, dysphagia is a significant but under-recognized complication of cardiac surgery. Advanced age, prolonged intubation, and surgical complexity are major risk factors. Standardized assessment protocols and early interventions are crucial to mitigating its impact and improving patient clinical outcomes.

## 1. Introduction

Dysphagia, or difficulty in swallowing, is a multifaceted clinical condition that can significantly impact quality of life (QoL) and lead to severe complications, such as aspiration pneumonia, malnutrition, and prolonged hospitalization [1,2,3]. Moreover, dysphagia after cardiac operations is associated with increased length of stay (LoS) and higher healthcare costs [4]. Post-operative dysphagia (PoD) is particularly concerning in the context of cardiac surgery due to the complexity and high-risk nature of these procedures. The incidence of dysphagia in this population is reported to vary widely, reflecting differences in surgical techniques, patient demographics, and diagnostic criteria [5,6,7]. Understanding the prevalence and underlying risk factors of dysphagia after cardiac surgery is critical for early identification, prevention, and management of dysphagia and its consequences.

Cardiac surgery, including procedures such as coronary artery bypass grafting (CABG), valve replacement, and aortic surgery, involves risks of neurological and anatomical disruptions [8,9]. Dysphagia in this context can arise from various factors, including intubation-related trauma, prolonged mechanical ventilation, ischemic brain injury, and post-operative edema. Moreover, pre-existing conditions such as advanced age, diabetes, or prior cerebrovascular disease may predispose patients to PoD [10,11,12]. Despite its clinical significance, PoD remains under-recognized and under-studied in cardiac surgical patients, with a lack of consensus on standardized assessment tools and risk stratification models.

This scoping review aims to consolidate current evidence on the incidence of dysphagia following cardiac surgery and to identify key risk factors contributing to its development.

## 2. Materials and Methods

### 2.1. Search Strategy and Criteria of Studies

This scoping review was conducted in accordance with the PRISMA-ScR guidelines [13]. This scoping review was registered in the Open Science Framework (https://doi.org/10.17605/OSF.IO/DUFGY). For the purpose of the investigation, a search was conducted in PubMed, Embase, Web of Sciences, and PEDro databases in order to find observational clinical studies that assessed the PoD occurrence and the factors associated with the presence of dysphagia in patients after cardiac surgery.

Studies included in this review met the following criteria: (1) prospective or retrospective clinical observational studies investigating the incidence of dysphagia and factors associated with PoD following cardiac surgery; (2) published in the English language; (3) including adult patients of both sexes and every nationality with age >18 years, undergoing cardiac surgery. Exclusion criteria comprised the following: (1) patients younger than 18 years old; (2) pre-existing dysphagia reported in the patient’s medical records or after the initial assessment before surgery; (3) neurological disorders such as dementia, Parkinson’s disease or syndrome, motor neuron disease, and recent ischemic stroke within the previous 3 months; (4) cancer and pre-existing head and neck surgery; and (5) patients with tracheostomy prior to surgery.

The search was performed between November and December of 2024. Specifically, the search was performed with keywords organized into three groups (participants, interventions, and results). The keywords used and their combinations during the search included the following: “(dysphagia OR deglutition disorder OR swallowing disorder) AND (cardiac surgery OR CABG OR valve surgery OR aortic surgery OR coronary artery bypass graft)”. These terms were chosen as umbrella terms and, thus, allowed us to capture as many published studies as possible.

Duplicates were removed from the initial total studies and the rest were reviewed twice. Firstly, two independent reviewers (C.K. and V.L.) screened titles, abstracts, and full texts according to the eligibility criteria. The final evaluation of the process was confirmed by a third independent investigator (S.D.). Discrepancies were resolved by consensus, and inter-rater agreement was assessed using Cohen’s kappa, which showed substantial agreement (κ = 0.78).

### 2.2. Extracted Primary and Secondary Clinical Outcomes

The primary clinical outcome of this review extracted from the included studies was the incidence figure of dysphagia in patients after cardiac surgery. Dysphagia as a clinical outcome could be determined by either subjective or objective outcome clinical instruments (bedside, imaging investigation, etc.). The secondary clinical outcome was the risk factors influencing the occurrence of dysphagia such as age, sex, BMI, diagnosis, type of surgery, duration of anesthesia, duration of aortic occlusion, surgical re-exploration, duration of mechanical ventilation, sedation and ICU stay, delusional disorder, hemodynamic instability, and/or use of vasoconstrictors/inotropes.

Risk factors were separated into mechanical, neurological, and procedural factors. Mechanical factors included prolonged intubation, direct laryngeal trauma, oropharyngeal edema, and aspiration risk. Neurological factors included cerebrovascular events, intra-operative hypoperfusion, and post-operative delirium. Procedural factors included prolonged cardiopulmonary bypass, use of intra-operative TEE, and surgical complexity (e.g., aortic or combined CABG–valve procedures).

## 3. Results

### 3.1. Search Results

**Figure 1:**
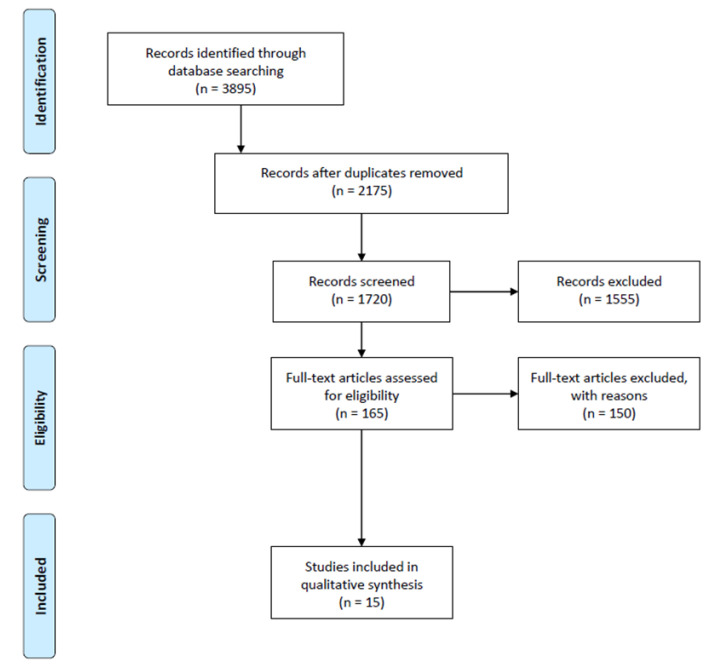
PRISMA-ScR flowchart of the included studies in this scoping review.

The initial search identified 3895 articles from the PubMed, Embase, Web of Sciences, and PEDro databases. The removal of duplicate publications and the title and abstract screening excluded 3730 articles. Among the 165 eligible articles, 140 of them either did not meet the inclusion criteria or were irrelevant to the subject, and 10 of them measured different clinical outcomes than those we were investigating. After the evaluation, 15 studies were finally included in our scoping review [4,5,6,7,14,15,16,17,18,19,20,21,22,23,24]. Search and screening results are demonstrated in the PRISMA-ScR flowchart (Figure 1).

The scoping review analyzed 15 studies evaluating the incidence and risk factors for dysphagia following cardiac surgery, revealing variations in prevalence and contributing factors.

Detailed baseline clinical characteristics and risk factors for dysphagia as well as the interventions, outcomes, and results of each study are presented in Table 1 and Table 2, respectively.

### 3.2. Incidence of Dysphagia Across Studies

The incidence of dysphagia varied widely, ranging from 2.7% [15] to 60% [19]. Nine studies utilized imaging and/or bedside swallowing assessments, while six reported dysphagia based on the bedside examination of swallowing or reporting of food management scales. Studies employing detailed objective assessments, such as fiberoptic endoscopic evaluation of swallowing (FEES) or videofluoroscopic swallow studies (VFSS), reported higher incidences compared to studies with bedside assessments only. Notably, patients undergoing non-emergent or combined surgeries, such as CABG with valve procedures, demonstrated higher rates of dysphagia. This variability underscores the importance of standardizing dysphagia diagnostic protocols in post-operative care and perhaps the need for formal imaging assessment of swallowing in this population.

### 3.3. Risk Factors Related to Patient Characteristics

Advanced age emerged as a consistent predictor of dysphagia across multiple studies [14,18,22]. Male gender [7,16] and pre-operative frailty [22,23] were also associated with increased risk. Comorbidities such as chronic lung disease, cerebrovascular disease, and reduced left ventricular ejection fraction (LVEF) further amplified this risk [15,16].

### 3.4. Procedural and Intra-Operative Risk Factors

The type and duration of surgical interventions were shown to be significant parameters for the presence of PoD. Prolonged intubation duration was significantly associated with higher dysphagia risk [5,20]. Specific procedures, such as aortic surgery [22], and intra-operative factors, including circulatory arrest and transesophageal echocardiography (TEE) usage, were independently linked to increased dysphagia rates [14,16].

### 3.5. Post-Operative Complications and Prognosis

Dysphagia was strongly associated with adverse clinical outcomes, including aspiration pneumonia [17,19], extended hospital stays [4], and increased healthcare costs [7]. Silent aspiration was prevalent, emphasizing the need for early and thorough assessments by speech–language pathologists [17,19]. Post-extubation dysphagia (PED) was particularly associated with frailty and post-operative complications, such as pneumonia and renal failure, impacting long-term recovery [22,23].

## 4. Discussion

This scoping review identified significant variability in the reported incidence of dysphagia following cardiac surgery, ranging from 2.7% to 60%. This broad range reflects differences in the patient populations, study designs, and methods of dysphagia assessment. Dysphagia was consistently associated with advanced age, prolonged intubation, cerebrovascular events, and specific surgical interventions such as aortic and combined CABG–valve procedures. Furthermore, dysphagia consequences were found to significantly delay recovery, increase LOS, and elevate healthcare costs due to secondary complications such as pneumonia and aspiration.

The wide range in dysphagia incidence could be attributed to several factors. Variability in the diagnostic criteria and methods of assessment plays a significant role. For instance, studies utilizing objective and sensitive techniques, such as VFSS or FEES [5,6,16,17,19,21], reported higher dysphagia rates compared to those relying on bedside assessments or self-reported symptoms [4,7,18,22,23,24]. Additionally, patient demographics and comorbidities differed across studies. Older patients with pre-existing conditions, such as frailty, chronic lung disease, and cerebrovascular disease, were more likely to develop dysphagia. Differences in surgical practices, including duration of the operation and the use of TEE, also contributed to variations in incidence. The inconsistency in defining dysphagia, whether based on instrumental findings or bedside assessments, further complicated direct comparisons.

The development of dysphagia following cardiac surgery is multifactorial, involving both mechanical and neurological components [4,6,21]. Prolonged intubation and the use of large-diameter endotracheal tubes can cause laryngeal trauma, vocal fold dysfunction, and edema, impairing swallowing mechanics [25,26]. The use of TEE during surgery has been implicated in oropharyngeal nerve injury due to prolonged pressure or ischemia [27,28]. Cerebrovascular events, a common complication in cardiac surgery, can disrupt central neural control of swallowing, leading to dysphagia [5]. Post-operative factors, such as prolonged ventilatory support, malnutrition, and aspiration, further exacerbate swallowing dysfunction [29,30,31]. Additionally, frailty and pre-existing neuromuscular impairments amplify the risk, particularly in older patients [32,33].

Advanced age was a universal risk factor, with studies consistently showing that older patients are more prone to dysphagia [4,7,14,18,22,23]. Prolonged intubation emerged as another critical risk factor, with studies demonstrating a direct association between intubation duration and dysphagia incidence [5,14,15,20,21]. Patients requiring extended ventilation support often experience oropharyngeal muscle deconditioning, increasing their susceptibility to aspiration [34,35]. Other significant predictors included pre-operative frailty [22,23,24], cerebrovascular disease [5,15,16,17,20,21], low BMI [4,16], and chronic lung conditions [16,19], highlighting the importance of a pre-operative risk assessment. Surgical factors, such as aortic or combined procedures and extended cardiopulmonary bypass times, also increased the likelihood of dysphagia, suggesting that procedural complexity and duration play significant roles [7,16,22,23].

Transesophageal echocardiography (TEE) is frequently utilized during cardiac surgery for intra-operative monitoring and assessment. However, its potential role as a risk factor for post-operative dysphagia remains underexplored. In our scoping review, three studies explicitly evaluated the association between TEE and dysphagia, with two identifying a significant correlation between TEE use and increased dysphagia incidence [14,15]. These findings align with previous reports suggesting that prolonged TEE probe placement may contribute to oropharyngeal and laryngeal trauma, transient nerve injury, or pharyngeal edema, all of which can impair swallowing function [27]. Notably, other included studies did not report on TEE use, which may either indicate routine application without documentation or a lack of consideration in their analyses. Additionally, studies with a higher proportion of TEE use did not consistently report increased dysphagia incidence, suggesting that factors such as probe duration, insertion technique, and patient susceptibility may play a role. Given the potential impact of intra-operative TEE on swallowing function, future research should aim to standardize the reporting of TEE usage and assess its independent contribution to dysphagia risk, particularly in high-risk populations undergoing prolonged cardiac procedures.

While several studies identified advanced age, prolonged intubation, and cerebrovascular events as significant predictors of dysphagia, other variables, such as gender, body mass index (BMI), and specific surgical techniques did not consistently demonstrate statistical significance across the included studies. This variability can be attributed to multiple factors, including sample size differences, study design heterogeneity, and variations in statistical power. For instance, some studies may have lacked sufficient sample sizes to detect a true association between certain risk factors and dysphagia, while others may not have adjusted for potential confounders, leading to inconsistent findings. Additionally, variations in diagnostic criteria and dysphagia assessment methods may have influenced which risk factors emerged as significant. For example, studies employing instrumental evaluations such as VFSS or FEES were more likely to identify subclinical dysphagia, potentially altering the observed associations with certain risk factors. Furthermore, some variables—such as intra-operative factors (e.g., duration of cardiopulmonary bypass, use of vasopressors, or TEE)—may contribute to dysphagia in select patient subgroups rather than the overall cardiac surgery population, making their effects less apparent in broad analyses. These discrepancies underscore the need for standardized study methodologies and larger, multicenter cohorts to more accurately determine the role of these variables in post-operative dysphagia.

Recurrent laryngeal nerve (RLN) injury is a well-recognized complication of cardiac surgery and has been implicated as a potential contributor to post-operative dysphagia [36]. The RLN, a branch of the vagus nerve, provides motor innervation to most intrinsic muscles of the larynx, playing a critical role in airway protection and swallowing function. Intra-operative factors such as prolonged intubation, direct surgical trauma during aortic arch or valve procedures, excessive retraction, or the use of transesophageal echocardiography (TEE) can result in transient or permanent RLN dysfunction [37]. This may lead to vocal cord paralysis, impaired airway protection, and aspiration risk, all of which exacerbate dysphagia [38]. Studies have shown that RLN injury is associated with a higher incidence of silent aspiration, prolonged ICU stays, and an increased need for speech and swallowing rehabilitation post-operatively [39,40]. Despite its clinical relevance, many studies do not systematically assess RLN function post-operatively, leading to underestimation of its role in dysphagia. Future research should incorporate standardized laryngeal assessments, such as laryngoscopy, to evaluate RLN injury in patients with post-cardiac surgery dysphagia, thereby improving risk stratification and guiding early interventions.

The clinical implications of dysphagia after cardiac surgery are profound, with studies reporting increased rates of pneumonia, prolonged hospital stays, and higher readmission rates [6,17,23,24]. Silent aspiration was a common finding, often leading to undiagnosed complications that further delayed recovery [5,17]. Dysphagia also resulted in substantial healthcare costs, driven by the need for additional interventions such as enteral feeding, speech and language therapy, and treatment of secondary complications like sepsis and renal failure [4]. Early identification and proactive management could reduce this burden, emphasizing the need for standardized post-operative care protocols [41,42].

Despite its comprehensive nature, this review has several limitations. The heterogeneity of included studies, encompassing both retrospective and prospective designs, limits the generalizability of findings. Many studies lacked standardized diagnostic criteria for dysphagia, leading to inconsistent reporting. The reliance on single-center data in some studies further restricts the applicability of findings to broader populations. Additionally, long-term clinical outcomes of dysphagia, such as persistent swallowing dysfunction and its impact on quality of life, were not uniformly assessed, leaving a critical gap in understanding the full scope of this complication.

Future studies should focus on standardizing dysphagia assessment methods to enable consistent and reliable comparisons across studies. Developing and validating risk stratification tools, such as the Risk of Dysphagia in Cardiac Surgery (RODICS) score, could facilitate early identification of high-risk patients [16,20]. Research should also explore the role of pre-operative interventions, including nutritional optimization and frailty reduction strategies, in minimizing dysphagia risk [20]. Long-term follow-up studies are needed to evaluate the persistence of dysphagia and its impact on patients’ functional status and quality of life. Additionally, cost-effectiveness analyses of preventative and rehabilitative measures would provide valuable insights for healthcare systems.

## 5. Conclusions

In conclusion, this review underscores dysphagia as a significant complication of cardiac surgery, with considerable variability in incidence due to differences in assessment methods and patient populations. The pathophysiology involves a complex interplay of mechanical, neurological, and procedural factors, with advanced age, prolonged intubation, and cerebrovascular events being the most consistent risk factors. Dysphagia significantly affects recovery, prolongs hospital stays, and increases healthcare costs. Addressing these challenges through standardized peri-operative assessment, early intervention, and comprehensive management strategies is essential to improve patient clinical outcomes and optimize resource utilization.

## Figures and Tables

**Table 1:** Patients’ baseline characteristics and risk factors for dysphagia: A summary of parameters assessed across studies in this scoping review.

Study	Year	Type of Study	Number of Patients (N)	Males, *n* (%)	Age (Years)	BMI (kg/m^2^)	Type of Surgery	Parameters of Interest
Hogue CW Jr et al. [14]	1995	Prospective cohort study	Total: 838 SD: 34 No-SD: 835	Total: 557 (66.5) SD: 20 (58.8) No-SD: 537 (64.3)	SD: 71.0 ± 2.0 No-SD: 63.0 ± 0.4	NA	SD: CABG 21 (62%), CABG/valvular procedure 3 (9%), arrhythmia surgery 2 (6%), valvular surgery 1 (3%), other 7 (21%) No-SD: CABG 621 (75%), CABG/valvular procedure 40 (5%), arrhythmia surgery 47 (6%), valvular surgery 35 (4%), other 90 (11%)	Age Duration of tracheal intubation TEE used during operation
Rousou JA et al. [15]	2000	Prospective cohort study	Total: 838 TEE: 126 No-TEE: 712	Total: 561 (67) TEE: 79 (62.7) No-TEE: 412 (67.7)	TEE: 66.3 ± 12 No-TEE: 66.9 ± 0.4	NA	TEE: CABG 39.7%, Non-CABG 60.3% No-TEE: CABG 79.3%, Non-CABG 20.7%	Stroke LVEF TEE Intubation time Operating room time
Barker J et al. [5]	2009	Retrospective study	Total: 254 DYS: 130 No-DYS: 124	Total: 176 (69.3) DYS: 91 (70.0) No-DYS: 85 (68.5)	Total: 64.5 ± 12.6 DYS: 65.4 ± 12.1 No-DYS: 63.5 ± 13.0	NA	Total: CABG 123 (48.4%), valve 59 (23.2%), other 72 (28.3%) DYS: CABG 57 (43.8%), valve 37 (28.5%), other 36 (27.7%) No-DYS: CABG 66 (53.2%), valve 22 (17.7%), other 36 (29.0%)	Duration of endotracheal intubation (hours) Type of procedure Urgency Peri-operative complications such as sepsis and stroke Intra-aortic balloon pump Low-output syndrome Number of endotracheal intubation events Hospital LOS (days) Post-extubation parameters
Grimm JC et al. [16]	2015	Prospective cohort study	Total: 1314 DYS: 115 No-DYS: 1199	Total: 852 (64.8) DYS: 83 (72.2) No-DYS: 769 (64.1)	DYS: 65 (53–74) * No-DYS: 61 (52–71) *	DYS: 27.0 ± 7.1 No-DYS: 29.4 ± 7.3	DYS: CABG 23 (20.0%), valve 24 (20.9%), VAD or heart transplant 17 (14.8%), other 6 (5.2%), combination 45 (39.1%) No-DYS: CABG 392 (32.7%), valve 234 (19.5%), VAD or heart transplant 57 (4.8%), other 70 (5.8%), combination 446 (37.2%)	Intra-operative (procedure, circulatory arrest, operative time in hours, bypass time in minutes) Post-operative (hospital LOS in days, ventilatory time (hours), reintubation, stroke, pneumonia, renal failure, in-hospital mortality) Age BMI Male CHF NYHA class
Daly E et al. [17]	2016	Retrospective study	Total: 190 DYS: 33 No-DYS: 157	NA	Total: 61.3 ± 15.0 DYS: 63.5 ± 17.6 No-DYS: 61.2 ± 14.4	NA	Total: CABG 99 (52.1%), valve 58 (30.5%), cardiac transplant 9 (4.7%), other 24 (12.6%) DYS: CABG 18 (54.5%), valve 10 (30.3%), cardiac transplant 1 (3.0%), other 4 (12.1%) No-DYS: CABG 81 (51.6%), valve 48 (30.6%), cardiac transplant 8 (5.1%), other 20 (12.7%)	Procedure Urgency Bypass time (hours) Intra-operative and post-operative TOE Total ventilation time (hours) Peri-operative complications such as stroke and sepsis Post-operative stroke History of stroke Tracheostomy Pneumonia Hospital LOS (days) Opiates after oral intake (days) Vocal fold paralysis Vocal fold edema Destination after hospital
Bowles BJ et al. [18]	2016	Prospective cohort study	176	132 (75.0)	73.5 ± 5.2	NA	CABG 96 (54.5), aortic valve replacement 20 (11.4), mitral valve repair or replacement 11 (6.3), CABG + single valve 23 (13.1), other 26 (14.8)	Age Gender Comorbidities (hypertension, diabetes mellitus, acute myocardial infarction, COPD, congestive heart failure, peripheral vascular disease, chronic renal insufficiency, chronic renal failure, aspiration)
Nguyen S et al. [4]	2016	Prospective cohort study	Total: 354 DYS: 56 No-DYS: 298	230 (65)	DYS: 73 ± 12.5 No-DYS: 64 ± 13.6	DYS: 25.0 ± 4.7 No-DYS: 27.6 ± 6.0	Non-emergent, non-transplant cardiac operations	Intubation time (minutes) ICU LOS (days) Hospital LOS (days) Readmission <30 days CVA Total costs (pharmacy and IV, room, surgical or medical supplies, cardiology, imaging and echo, respiratory therapy) Pneumonia In-hospital mortality
Miles A et al. [19]	2018	Retrospective study	106	72 (69)	63 ± 15.16	NA	CABG 59 (56%), valve 37 (35%), cardiac transplant 2 (2%), other (infection, aneurysm) 8 (8%)	Pre-operative (age, gender, ethnicity, NYHA score, previous stroke, EuroSCORE II) Peri-operative (procedure, ventilation time, stroke) Post-operative (died during admission, tracheostomy, duration of tracheostomy, pneumonia, enteral feeding duration, length of stay in hospital, destination on discharge, readmissions within 3 months of discharge, vocal fold paralysis, vocal fold edema, laryngeal mucosal changes, tracheal stenosis)
Zhou XD et al. [20]	2019	Prospective cohort study	395	258 (73)	61.7 ± 12.8	23.4 ± 3.3	CABG	Length of endotracheal intubation (hours) Length of gastric intubation (hours) Sedative drug use duration (hours) Previous stroke
Black RJ et al. [21]	2019	Retrospective study	Total: 284 SP: 68 No-SP: 216	Total: 159 (56) SP: 40 (58.8) No-SP: 119 (55.1)	Total: 46.7 ± 14.1 SP: 48.3 ± 13.0 No-SP: 46.2 ± 14.4	NA	Total: bilateral lung transplant 175 (62%), heart transplant 101 (36%), single lung transplant 4 (1%), heart and lung transplant 4 (1%) SP: bilateral lung transplant 42 (62%), heart transplant 25 (37%), heart and lung transplant 1 (1%)	Pre-operative factors (age, gender, respiratory disease, history of chronic heart failure, chronic heart failure at admission, history of smoking, CVA, cardiac arrhythmia, hypertension, diabetes, pulmonary hypertension, diabetes–insulin-requiring, cardiac angina, diabetes–insulin-dependent, cerebrovascular disease, chronic renal failure) Post-operative (total hours intubated, times intubated, ICU LOS (days), number of admissions in the ICU)
Ogawa M et al. [22]	2022	Retrospective cohort study	Total: 644 PED: 98 No-PED: 566	Total: 382 (57.5) PED: 52 (53.1) No-PED: 330 (58.3)	Total: 67.72 ± 13.69 PED: 73.62 ± 9.78 No-PED: 66.55 ± 14.12	Total: 22.86 ± 3.79 PED: 22.30 ± 3.71 No-PED: 22.97 ± 3.80	Total: aortic 88 (13.3%), CABG 88 (13.3%), valve 434 (65.4%), combination 54 (8.1%) PED: aortic 27 (27.6%), CABG 9 (9.2%), valve 52 (53.1%), combination 10 (10.2%) No-PED: aortic 61 (10.8%), CABG 79 (14.0%), valve 382 (67.5%), combination 44 (7.8%)	Age Female gender Previous cardiac surgery eGFR Hemoglobin MNA-SF, at risk and/or malnutrition Frailty NYHA functional class EuroSCORE II Type of surgery Ventilation time
Verma A et al. [7]	2022	Retrospective cohort study	Total: 2,319,628 LC: 39,688 No-LC: 2,279,940	Total: 1,603,477 (69.1) LC: 25,758 (64.9) No-LC: 1,577,719 (69.2)	LC: 72 (63–79) * No-LC: 67 (59–74) *	NA	LC: CABG 52.1%, valve 26.4%, CABG + valve 17.4%, multiple valve 4.0% No-LC: CABG 61.5%, valve 23.8%, CABG + valve 11.9%, multiple valve 2.8%	Pre-operative (age, gender, Elixhauser Index, elective admission, income, insurance type, operation type, comorbidities, hospital volume, teaching status) Complications including neurologic, cardiac, thrombotic, and infectious Outcomes including in-hospital mortality, pneumonia, prolonged ventilation, reintubation, tracheostomy, non-home discharge, 30-day non-elective readmission, length of stay in days, and cost
Plowman EK et al. [6]	2023	Prospective cohort study	Total: 182 ASP: 53 No-ASP: 129	Total: 122 (67) ASP: 39 (74) No-ASP: 83 (64)	Total: 62.3 ± 13.3 ASP: 61.8 ± 12.8 No-ASP: 63.4 ± 14.6	Total: 29.7 ± 6.3 ASP: 29.9 ± 6.4 No-ASP: 29.2 ± 6.0	Total: CABG or valve 72, aortic root 12, arch 40, LVAD or transplant 9, arch and valve 43, other 6 ASP: CABG or valve 46, aortic root 10, arch 26, LVAD or transplant 6, arch and valve 26, other 4 No-ASP: CABG or valve 26, aortic root 2, arch 14, LVAD or transplant 3, arch and valve 17, other 2	Demographics (age, gender, ethnicity, smoking, stroke, diabetes mellitus, obstructive sleep apnea, RSI score, gastroesophageal reflux, EuroSCORE II, NYHA, ASA) Endotracheal tube (intubation duration, multiple intubations, ETT size, Hi–Lo Evac ETT, single- or double-lumen ETT) Operative (reoperative surgery, urgency, type of surgery, circulatory arrest time, sedation time, surgery time, TEE time, TEE images captured, crossclamp time, cardiopulmonary bypass time)
Ogawa M et al. [23]	2023	Retrospective cohort study	Total: 712 Abnormal PED: 104 No-PED: 608	Total: 408 (57.5) PED: 55 (52.9) No-PED: 353 (58.1)	Total: 67.7 ± 13.7 PED: 73.5 ± 9.8 No-PED: 66.8 ± 14.0	PED: 22.3 ± 3.6 No-PED: 22.9 ± 3.8	Total: CABG 58 (9.6%), valve 384 (63%), aortic, combination PED: CABG 11 (10.6%), valve 55 (52.9%), aortic 27 (26%), combination 11 (10.6%) No-PED: CABG 84 (13.8%), valve 411 (67.6%), aortic 61 (10.8%), combination 52 (8.6%)	Operative time in hours Ventilation time in hours Procedure type Pneumonia Infection-related complications Renal failure ICU stay in days Hospital stay in days Discharge location Hospital-associated disability
Ogawa M et al. [24]	2024	Retrospective cohort study	Total: 442 Abnormal MPT: 243 Normal MPT: 199	Total: 247 (56) Abnormal MPT: 132 (54) Normal MPT: 115 (58)	Total: 71.5 ± 6.4 Abnormal MPT: 72.8 ± 6.5 Normal MPT: 69.9 ± 6.0	Total: 22.7 ± 3.8 Abnormal MPT: 22.7 ± 3.9 Normal MPT: 22.7 ± 3.8	Total: CABG 58 (9.6%), valve 384 (63%) Abnormal MPT: CABG 32 (9.5%), valve 211 (62%) Normal MPT: CABG 26 (9.7%), valve 173 (64%)	Age Male gender BMI Type of surgery NYHA functional class MPT Frail MNA-SF EuroSCORE II

BMI, body mass index; SD, swallowing dysfunction; TEE, transesophageal echocardiography; CABG, coronary artery bypass grafting; LVEF, left ventricular ejection fraction; DYS, dysphagia; LOS, length of stay; CHF NYHA, chronic heart failure New York Heart Association; TOE, transesophageal echocardiogram; COPD, chronic obstructive pulmonary disease; SP, speech pathology; PED, post-extubation dysphagia; ASP, aspirators; RSI, Reflux Symptom Index; EuroSCORE, European System for Cardiac Operative Risk Evaluation; ASA, American Society of Anesthesiologists; ETT, endotracheal tube; LVAD, left ventricular assist device; GERD, gastroesophageal reflux disease; LC, laryngeal complications; MPT, maximum phonation time; MNA-SF, Mini Nutritional Assessment Short-Form; EuroSCORE, European System for Cardiac Operative Risk Evaluation; NA, not available. * Expressed in median (IQR).

**Table 2:** Population, intervention, comparison, and outcomes of each study included in this scoping review. Table is separated into imaging investigated and bedside assessment of dysphagia.

Study	Method of Assessment	Patients with Dysphagia, *n* (%)	Type of Dysphagia	Prognostic Factors of Dysphagia	Conclusions
Hogue CW Jr et al. [14]	Barium swallow cineradiography	34 out of 869 (4%)	Oropharyngeal	Age (*p* < 0.001) Length of tracheal intubation after the operation (*p* = 0.001) Intra-operative use of TEE (*p* = 0.003)	Dysfunctional swallowing after cardiac operations is more common in older patients. An association between intra-operative use of TEE and swallowing dysfunction was also observed.
Rousou JA et al. [15]	Barium swallow cineradiography	23 out of 838 (2.7%)	Oropharyngeal	Intubation time (*p* = 0.005; OR = 1.01, 95% CI 1.00–1.03) Stroke (*p* < 0.001; OR = 21.7, 95% CI 4.93–95.9) LVEF < 30% (*p* = 0.023; OR = 5.50, 95% CI 1.16–26.4) TEE (*p* < 0.001; OR = 7.80, 95% CI 1.81–33.6)	TEE may be an independent risk factor for dysphagia following cardiac operations.
Barker J et al. [5]	A speech–language pathologist conducted a full swallowing assessment at the bedside in addition to a more objective videofluoroscopic assessment if required.	130 out of 254 (51%)	Oropharyngeal	Duration of endotracheal intubation (*p* < 0.001) Occurrence of a peri-operative cerebrovascular event (*p* = 0.014) Presence of peri-operative sepsis (*p* = 0.016)	Dysphagia is more common in patients with prolonged endotracheal intubation after cardiac surgery than has previously been reported. The duration of post-operative endotracheal intubation is a strong predictor of subsequent dysphagia that both prolongs the return to normal oral feeding and delays subsequent hospital discharge. Patient- or procedure-specific factors are not good predictors. To accelerate discharge of high-risk patients, aggressive nutritional supplementation should pre-empt extubation and swallowing surveillance should follow.
Grimm JC et al. [16]	Videofluoroscopic swallow study (VFSS)	115 out of 1314 (8.8%)	Oropharyngeal	Male (*p* = 0.04; OR = 1.66, 95% CI 1.04–2.65) BMI < 20 kg/m^2^ (*p* = 0.01; OR = 3.02, 95% CI 1.33–6.88) Chronic lung disease (*p* < 0.001; OR = 2.36, 95% CI 1.48–3.75) Cerebrovascular disease (*p* = 0.01; OR = 2.24, 95% CI 1.28–3.91) Procedure—VAD or heart transplant (*p* < 0.001; OR = 3.18, 95% CI 1.45–6.94) Circulatory arrest (*p* = 0.01; OR = 4.41, 95% CI 1.58–12.36) Ventilation >24 h (*p* < 0.001; OR = 4.23, 95% CI 2.74–6.54)	The incidence and impact of dysphagia after open cardiac operations are significant. The Risk of Dysphagia in Cardiac Surgery (RODICS) score could lead to prompt identification of patients at high risk for post-operative dysphagia and potentially minimize the complications of aspiration.
Daly E et al. [17]	Speech–language pathologist by bedside assessment and/or instrumental assessment (FEES or VFSS)	33 out of 190 (17.4%)	Oropharyngeal	Post-operative stroke (*p* < 0.001; OR = 7.14, 95% CI 1.79–28.40) History of stroke (*p* < 0.01; OR = 3.34, 95% CI 1.01–10.91) Tracheostomy (*p* < 0.01; OR = 2.50, 95% CI 1.04–5.99)	Patients identified with dysphagia after cardiac surgery had a high incidence of silent aspiration and increased risk of pneumonia. Early identification and ongoing assessment and appropriate management of dysphagic patients by a speech–language pathologist are strongly recommended.
Nguyen S et al. [4]	FEES performed by inpatient speech and language pathologist (SLP)	56 out of 354 (16%)	Oropharyngeal	Patients with higher age, lower BMI, lower LVEF, anemia, prior history of CHF, and longer operative times presented dysphagia	Dysphagia is an independent and major contributor to health care costs after cardiac operations, suggesting that post-operative dysphagia represents a highly suitable target for quality improvement and cost containment efforts.
Miles A et al. [19]	FEES DOSS (1 = severe dysphagia; while 7 = nil) FOIS (scores 1–3 indicate enteral feeding; while 7 indicates a non-modified diet)	64 out of 106 (60%) patients were classified as severe or moderate–severe dysphagia	Oropharyngeal	Length of stay (R−0.33, *p* < 0.01) Nasogastric feed duration (R−0.46, *p* < 0.001) Tracheostomy duration (R−0.36, *p* < 0.001) Pneumonia (rpb−0.32, *p* < 0.01) Model including age, cardiac procedure, ventilation time, tracheostomy tube duration, and stroke (pre- or post-surgery) [x2 4.89, *p* < 0.05] explained 62% (Nagelkerke R2) of the variance in vocal fold motion impairment	Early endoscopic assessment for identification of dysphagia and laryngeal injury in patients following cardiothoracic surgery may allow for early management and prevention of secondary complications.
Black RJ et al. [21]	Standard bedside assessment (BSA) and/or an objective swallowing assessment via videofluoroscopy or FEES	58 out of 66 (88%) who were referred to SP	Oropharyngeal	Cerebrovascular disease (*p* = 0.032; OR = 6.747, 95% CI 1.179–38.601) Intubations (*p* = 0.028; OR = 2.066, 95% CI 1.081–3.951) Intubation duration (*p* < 0.001; OR = 1.004, 95% CI 1.002–1.007) Stay in the ICU (*p* < 0.001; OR = 1.068, 95% CI 1.046–1.094) Admissions to the ICU (*p* = 0.046; OR = 1.384, 95% CI 1.005–1.906)	Significant clinical indicators for referral to SP for the management of oropharyngeal dysphagia and laryngeal dysfunction in patients after lung or heart transplantation.
Plowman EK et al. [6]	FEES performed by a trained speech–language pathologist	95 out of 182 (52%) clinically significant pharyngeal residue	Oropharyngeal	TEE images > 110 [2.6 (1.1–6.3)] NYHA ≥ III [2.9 (1.2–7.0)] ETT ≥ 8.0 [3.1 (1.1–8.6)]	Tracheal aspiration was prevalent, covert, and associated with increased morbidity and mortality.

BMI, body mass index; TEE, transesophageal echocardiography; CABG, coronary artery bypass grafting; LVEF, left ventricular ejection fraction; CHF NYHA, chronic heart failure New York Heart Association; SP, speech pathology; PED, post-extubation dysphagia; ETT, endotracheal tube; LC, laryngeal complications; MPT, maximum phonation time; EuroSCORE, European System for Cardiac Operative Risk Evaluation; DOSS, Dysphagia Outcome and Severity Scale; FOIS, Functional Oral Intake Scale; VFSS, videofluoroscopic study of swallowing; LVH, low volume hospital; MVH, medium volume hospital; HVH, high volume hospital; HAD, hospital-associated disability.

## Data Availability

No new data were created or analyzed in this study.

## Abbreviations

The following abbreviations are used in this manuscript:

QoL	Quality of life
LoS	Length of stay
PoD	Post-operative dysphagia
CABG	Coronary artery bypass grafting
FEES	Fiberoptic endoscopic evaluation of swallowing
VFSS	Videofluoroscopic swallow studies
TEE	Transesophageal echocardiography
LVEF	Left ventricular ejection fraction
PED	Post-extubation dysphagia

## References

[1] Karunaratne T.B., Clavé P., Ortega O. (2024). Complications of oropharyngeal dysphagia in older individuals and patients with neurological disorders: Insights from Mataró hospital, Catalonia, Spain. Front. Neurol..

[2] Al Rjoob M., Hassan N.F.H.N., Aziz M.A.A., Zakaria M.N., Mustafar M.F.B.M. (2022). Quality of life in stroke patients with dysphagia: A systematic review. Tunis. Med..

[3] Song J.M. (2024). Dysphagia and quality of life: A narrative review. Ann. Clin. Nutr. Metab..

[4] Nguyen S., Zhu A., Toppen W., Ashfaq A., Davis J., Shemin R., Mendelsohn A.H., Benharash P. (2016). Dysphagia after cardiac operations is associated with increased length of stay and costs. Am. Surg..

[5] Barker J., Martino R., Reichardt B., Hickey E.J., Ralph-Edwards A. (2009). Incidence and impact of dysphagia in patients receiving prolonged endotracheal intubation after cardiac surgery. Can. J. Surg..

[6] Plowman E.K., Anderson A., York J.D., DiBiase L., Vasilopoulos T., Arnaoutakis G., Beaver T., Martin T., Jeng E.I. (2023). Dysphagia after cardiac surgery: Prevalence, risk factors, and associated outcomes. J. Thorac. Cardiovasc. Surg..

[7] Verma A., Hadaya J., Tran Z., Dobaria V., Madrigal J., Xia Y., Sanaiha Y., Mendelsohn A.H., Benharash P. (2022). Incidence and outcomes of laryngeal complications following adult cardiac surgery: A national analysis. Dysphagia.

[8] McDonagh D.L., Berger M., Mathew J.P., Graffagnino C., Milano C.A., Newman M.F. (2014). Neurological complications of cardiac surgery. Lancet Neurol..

[9] Gilbey T., Milne B., de Somer F., Kunst G. (2023). Neurologic complications after cardiopulmonary bypass—A narrative review. Perfusion.

[10] Mao L., Wang J., Li Y., Zheng J., Fan D., Wei S., Wu X., Yang X., Wang D. (2024). Risk factors for dysphagia in patients with acute and chronic ischemic stroke: A retrospective cohort study. Heliyon.

[11] Yang C., Pan Y. (2022). Risk factors of dysphagia in patients with ischemic stroke: A meta-analysis and systematic review. PLoS ONE.

[12] Regala M., Marvin S., Ehlenbach W.J. (2019). Association Between Postextubation Dysphagia and Long-Term Mortality Among Critically Ill Older Adults. J. Am. Geriatr. Soc..

[13] Tricco A.C., Lillie E., Zarin W., O’Brien K.K., Colquhoun H., Levac D., Moher D., Peters M.D.J., Horsley T., Weeks L. (2018). PRISMA Extension for Scoping Reviews (PRISMA-ScR): Checklist and Explanation. Ann. Intern. Med..

[14] Hogue C.W., Lappas G.D., Creswell L.L., Ferguson T.B., Sample M., Pugh D., Balfe D., Cox J.L., Lappas D.G. (1995). Swallowing dysfunction after cardiac operations. Associated adverse outcomes and risk factors including intraoperative transesophageal echocardiography. J. Thorac. Cardiovasc. Surg..

[15] Rousou J.A., Tighe D.A., Garb J.L., Krasner H., Engelman R.M., Flack J.E., Deaton D.W. (2000). Risk of dysphagia after transesophageal echocardiography during cardiac operations. Ann. Thorac. Surg..

[16] Grimm J.C., Magruder J.T., Ohkuma R., Dungan S.P., Hayes A., Vose A.K., Orlando M., Sussman M.S., Cameron D.E., Whitman G.J. (2015). A novel risk score to predict dysphagia after cardiac surgery procedures. Ann. Thorac. Surg..

[17] Daly E., Miles A., Scott S., Gillham M. (2016). Finding the red flags: Swallowing difficulties after cardiac surgery in patients with prolonged intubation. J. Crit. Care.

[18] Bowles B.J., Puntil-Sheltman J. (2016). Is dysphagia after cardiac operations a “preexisting condition”?. Ann. Thorac. Surg..

[19] Miles A., McLellan N., Machan R., Vokes D., Hunting A., McFarlane M., Holmes J., Lynn K. (2018). Dysphagia and laryngeal pathology in post-surgical cardiothoracic patients. J. Crit. Care.

[20] Zhou X.D., Dong W.H., Zhao C.H., Feng X.F., Wen W.W., Tu W.Y., Cai M.X., Xu T.C., Xie Q.L. (2019). Risk scores for predicting dysphagia in critically ill patients after cardiac surgery. BMC Anesthesiol..

[21] Black R.J., Bogaardt H., McCabe P., Glanville A.R., MacDonald P., Madill C. (2019). Clinical predictors for oropharyngeal dysphagia and laryngeal dysfunction after lung and heart transplantation. Int. J. Lang. Commun. Disord..

[22] Ogawa M., Satomi-Kobayashi S., Yoshida N., Komaki K., Izawa K.P., Hamaguchi M., Inoue T., Sakai Y., Hirata K.I., Okada K. (2022). Impact of frailty on postoperative dysphagia in patients undergoing elective cardiovascular surgery. JACC Asia.

[23] Ogawa M., Satomi-Kobayashi S., Hamaguchi M., Komaki K., Izawa K.P., Miyahara S., Inoue T., Sakai Y., Hirata K.I., Okada K. (2023). Postoperative dysphagia as a predictor of functional decline and prognosis after undergoing cardiovascular surgery. Eur. J. Cardiovasc. Nurs..

[24] Ogawa M., Satomi-Kobayashi S., Hamaguchi M., Komaki K., Kusu H., Izawa K.P., Miyahara S., Sakai Y., Hirata K.I., Okada K. (2024). Impact of maximum phonation time on postoperative dysphagia and prognosis after cardiac surgery. JTCVS Open.

[25] Colton House J., Noordzij J.P., Murgia B., Langmore S. (2011). Laryngeal injury from prolonged intubation: A prospective analysis of contributing factors. Laryngoscope.

[26] Shinn J.R., Kimura K.S., Campbell B.R., Sun Lowery A., Wootten C.T., Garrett C.G., Francis D.O., Hillel A.T., Du L., Casey J.D. (2019). Incidence and outcomes of acute laryngeal injury after prolonged mechanical ventilation. Crit. Care Med..

[27] Zhang L., Xie Y., Ren Z., Xie M. (2024). Transesophageal echocardiography related complications. Front. Cardiovasc. Med..

[28] Mathur S.K., Singh P. (2009). Transoesophageal echocardiography related complications. Indian J. Anaesth..

[29] Macht M., White S.D., Moss M. (2014). Swallowing dysfunction after critical illness. Chest.

[30] Rassameehiran S., Klomjit S., Mankongpaisarnrung C., Rakvit A. (2015). Postextubation dysphagia. Bayl. Univ. Med. Cent. Proc..

[31] Ambrosino N., Clini E. (2004). Long-term mechanical ventilation and nutrition. Respir. Med..

[32] Robison R., Focht Garand K.L., Affoo R., Yeh C.K., Chin N., McArthur C., Pulia M., Rogus-Pulia N. (2023). New horizons in understanding oral health and swallowing function within the context of frailty. Age Ageing.

[33] Yang R.Y., Yang A.Y., Chen Y.C., Lee S.D., Lee S.H., Chen J.W. (2022). Association between dysphagia and frailty in older adults: A systematic review and meta-analysis. Nutrients.

[34] Elpern E.H., Scott M.G., Petro L., Ries M.H. (1994). Pulmonary aspiration in mechanically ventilated patients with tracheostomies. Chest.

[35] Mirzakhani H., Williams J.N., Mello J., Joseph S., Meyer M.J., Waak K., Schmidt U., Kelly E., Eikermann M. (2013). Muscle weakness predicts pharyngeal dysfunction and symptomatic aspiration in long-term ventilated patients. Anesthesiology.

[36] Alfares F.A., Hynes C.F., Ansari G., Chounoune R., Ramadan M., Shaughnessy C., Reilly B.K., Zurakowski D., Jonas R.A., Nath D.S. (2016). Outcomes of recurrent laryngeal nerve injury following congenital heart surgery: A contemporary experience. J. Saudi Heart Assoc..

[37] Dimarakis I., Protopapas A.D. (2004). Vocal cord palsy as a complication of adult cardiac surgery: Surgical correlations and analysis. Eur. J. Cardiothorac. Surg..

[38] Hamdan A.L., Moukarbel R.V., Farhat F., Obeid M. (2002). Vocal cord paralysis after open-heart surgery. Eur. J. Cardiothorac. Surg..

[39] Wallace S., McGrath B.A. (2021). Laryngeal complications after tracheal intubation and tracheostomy. BJA Educ..

[40] Black R.J., Novakovic D., Plit M., Miles A., MacDonald P., Madill C. (2021). Swallowing and laryngeal complications in lung and heart transplantation: Etiologies and diagnosis. J. Heart Lung Transplant..

[41] Zhang X., Zhao J., Zheng L., Li X., Hao Y. (2022). Implementation strategies to improve evidence-based practice for post-stroke dysphagia identification and management: A before-and-after study. Int. J. Nurs. Sci..

[42] Rosenvinge S.K., Starke I.D. (2005). Improving care for patients with dysphagia. Age Ageing.

